# Frontiers and Challenges in Occupational Safety and Health

**DOI:** 10.3389/fpubh.2014.00085

**Published:** 2014-07-16

**Authors:** How-Ran Guo

**Affiliations:** ^1^Department of Environmental and Occupational Health, National Cheng Kung University, Tainan, Taiwan; ^2^Department of Occupational and Environmental Medicine, National Cheng Kung University Hospital, Tainan, Taiwan

**Keywords:** occupational safety, occupational health, nanomaterial, mental health, ergonomics, shiftwork, cardiovascular disease, health promotion

Most people work to make a living, but unfortunately many at the cost of their safety, health, and even lives. The International Labour Organization (ILO) estimated that each year poor occupational health and safety led to 271 million injuries, 160 million occupational diseases, and 2 million work-related deaths (1). Whereas, the development of occupational safety and health may be traced back to thousands of years, there are many areas that need to be explored and new challenges to be overcome.

## Global Coverage of Occupational Safety and Health

The most fundamental issue that needs to be addressed is that the occupational safety and health discipline does not even exist in many geographic areas, in spite of the generally accepted concept that the protection of the worker against sickness, disease, and injury arising out of employment is not only a labor right but also a fundamental human right (2). According to the estimation made by the ILO in 2003, only 10–15% of the total global workforce has access to some kind of occupational health services (1). While the ILO has 185 member states currently (3), the International Commission on Occupational Health (ICOH), the world’s leading international scientific society in the field of occupational health, has membership from 93 countries only (4), and the International Occupational Hygiene Association (IOHA), the world’s leading international scientific society in the field of industrial hygiene, has membership from 29 member organizations representing 27 countries only (5). Both ICOH and IOHA recognized that areas where occupational health (hygiene) is not recognized or organized are the same areas where there is the greatest need for occupational health services. These areas are mostly in the developing countries, and they are literally the frontiers in occupational safety and health.

## Nanomaterials and Particulate Matters

With advancement in industry, new chemicals are introduced into our work environment on a regular basis. In recent decades, however, “size” has become an important issue. While their chemical components vary widely, engineered nanoparticles have drawn a lot of attention as a single entity, simply because they are at the same level in terms of size. With the small sizes, they should be able to penetrate various barriers such as cell membranes and deposit in organelles such as mitochondria. They may also travel throughout the body and might cause injurious responses that humans have never encountered (6). A property closely related to the small size is the relatively large surface area, which is an important factor for toxic effects (6). Although epidemiological data on the adverse effects of nanoparticles on humans are limited, governmental agencies in some countries have raised the question of whether innocuous materials such as carbon may cause serious health outcomes such as cancer when they are in the form of nanoparticles (7). Shape, defect density, physicochemical stability, and surface modification are regarded as the main causes that elicit altered physiological response or cytotoxicity when the size of the same material is reduced to the nanolevel (8). It was recently estimated that the global market for nanomaterial-based products may reach 100 billion dollars per year for 2011–2015, and therefore, the occupational safety health issues related to their production need to be explored immediately (8). At a larger scale, heath effects of particulate matters (PMs) are better documented. In addition to cardiovascular and respiratory effects that are more readily linked to PMs (9), the International Agency for Research on Cancer (IARC) has classified PM as a Group 1 carcinogen (carcinogenic to humans) (10). PM_2.5_, the smallest group of the PMs, has drawn growing attention. Many diseases have been linked to PM_2.5_ exposures. In fact, some epidemiological studies have observed associations between PM_2.5_ exposures and mental disorders such as autism, not just physical illness (11, 12).

## Occupational Mental and Behavioral Disorders

While mental health has been a great concern in the workplace, it was not until 2010 that ILO included “Mental and Behavioral Disorders” in its *ILO List of Occupational Diseases* (13). It is listed under “Occupational diseases by target organ systems.” Unlike disorders of other organ systems, most mental and behavioral disorders do not have solid biological basis for diagnosis, except for those caused by chemicals and brain injuries. In fact, since the biological mechanism of most mental and behavioral disorders remains unclear, their diagnosis are mainly based on “criteria,” and it is hard to assess their associations with work. Post-traumatic stress disorder (PTSD) may be an exception, where a “stressor” is an essential diagnostic criterion (14). If the stressor was arose from work activities, the illness should be attributable to the occupation and be regarded as an occupational disease. While this logic seems simple, the complexity of the etiology of mental disorders had hindered the inclusion of this disease in the list till 2010. On the other hand, the relatively straightforward causal relationship has made PTSD the only specifically included item in this category of the list. Nonetheless, as in other categories, the list also includes an open item “other mental or behavioral disorders not mentioned in the preceding item where a direct link is established scientifically, or determined by methods appropriate to national conditions and practice, between the exposure to risk factors arising from work activities and the mental and behavioral disorder(s) contracted by the worker.” Although such an item is a new feature of the revised list and is added to every category, it implies the potential of including other mental and behavioral disorders in the future. Hopefully, with the better understanding of work-related PTSD, a mental disorder with well-defined etiology, we may achieve a breakthrough in the knowledge of mental and behavioral disorders, particularly on their mechanisms. One promising direction is the study of biomarkers on mental stress such as the saliva cortisol level (15), which link stressors to physiological responses.

## Shift Work and Burnout

With the improvement of lighting, our work schedule has been extended to night time. With the globalization, shift work has become an essential part of business in more industries, instead of just a measure to increase productivity. In the United States, more than 21 million (17.7%) wage and salary workers worked alternate shifts on a regular basis in 2004 (16), and in the European Union, up to 20% of the working population were involved in some form of shift work in 2007 (17). The numbers are expected to increase. Shift work is associated with many diseases, including sleeping disorder, metabolic syndrome, diabetes, cardiovascular disease, and cancer (18–20). In addition, it may increase injuries and threaten the safety of the workplace (20, 21). As shift work has become a necessary evil associated with globalization, occupational safety and health professionals need to identify the working schedules that are less harmful to the workers while meeting the business needs at the same time. An emerging problem associated with shift work is extended working hours. The term “karoshi” was first applied by Japanese to describe the most serious outcome of overwork death (22), but the concept of “burnout” has been around the medical field for a long time, especially in the health profession itself (23, 24). Whereas most such cases can be attributed to cardiovascular and cerebrovascular disorders (22, 25, 26), “burnout” has not been identified as a medical term scientifically. In fact, aside from “karoshi,” “burnout” is also used to cover mental illness related to overwork, which may be introduced by stressors other than long working hours (15). More studies are needed to construct the scientific basis of “burnout” and establish its diagnostic criteria and prevention strategy.

## Diagnostic Criteria for Ergonomic Disorders

The lack of diagnostic criteria is not limited to emerging occupational diseases like mental disorders. In most developed countries, back pain is the most common occupational disorder (27), and the majority of work-related back pain can be attributable to ergonomic hazards (28). When a developing country advances toward becoming a developed country, the service sector occupies a larger proportion of the workforce. As a result, physical, chemical, and biological hazards in the workplace become less prevalent, and ergonomic hazards become increasingly important. Although numerous studies have been conducted on back pain (27), data on the dose–response relationship between risk factors and occurrence of back pain are still limited, making objective determination of its association with work activities difficult. Other prevalent musculoskeletal disorders such as trigger fingers, carpal tunnel syndrome, tennis elbow, and rotator cuff syndrome have the same problem. When more countries are moving on the path to becoming a developed country, the construction of solid scientific bases for the diagnosis, and thus prevention, of occupational disorders caused by ergonomic hazards is an urgent need that has not yet been met (29).

## Health Promotion in the Workplace

While we generally face more health hazards and safety risks in the workplace than in the living environment, the workplace is also the best place to implement health promotion programs. For example, on the World Heart Day 2009, the World Heart Federation called on people to “Work with Heart”: take action in the workplace to improve health and productivity and reduce the risk of cardiovascular disease. This is because it recognized that “The workplace allows access to an estimated 54% of the world’s population providing the ideal platform to inspire individuals and groups…” and that “Workplace wellness programs have been shown to have many benefits, for both employes and employers.” (30). Apart from treating and preventing occupational diseases, promoting the general health status of workers through measures implemented in the workplace should be the ultimate goal of occupational safety and health. More studies should be carried out to identify the ways to achieve this goal more effectively.

## Conflict of Interest Statement

The author declares that the research was conducted in the absence of any commercial or financial relationships that could be construed as a potential conflict of interest.
